# Fine-scale plasticity in nest placement can compensate for poor quality junipers as nesting trees for gray vireos

**DOI:** 10.7717/peerj.12477

**Published:** 2021-11-09

**Authors:** Jonathan Harris, Loren Smith, Scott McMurry

**Affiliations:** Integrative Biology, Oklahoma State University, Stillwater, Oklahoma, United States

**Keywords:** Gray vireo, Vireo vicinior, Pinyon-juniper, Behavioral plasticity, Nest survival, Nest placement

## Abstract

Understanding the interactions between behavior and habitat characteristics can have important implications for species of conservation concern. Gray vireos (*Vireo vicinior*) are one example of a species of conservation concern that is understudied in terms of nest survival probabilities and the habitat characteristics that influence them. Our objective was to determine if habitat features such as juniper density, juniper foliage density, or tree height influence nest survival probabilities, and if gray vireo nest placement can mitigate habitat risks. Based on previous work, we expected daily nest survival probabilities to be associated with nest height and surrounding vegetation. We monitored 89 nests in central New Mexico from 2016–2018 to estimate daily nest survival probabilities. We compared variation in nest placement, nest tree characteristics, and surrounding vegetation between failed and successful nests using logistic exposure models and Akaike Information Criteria. Daily and cumulative nest survival probability were 0.983 (95% CI [0.973–0.989]) and 0.575 (95% CI [0.444–0.702]), respectively. Top models predicting nest survival included a negative interaction between nest-tree foliage density and the distance of the nest from the edge of the nesting tree. This suggests that gray vireos can mitigate risks associated with low nest concealment by nesting closer to the interior of the nesting tree.

## Introduction

Species can often mitigate predation risk through changes in behavior (Lima & Dill, 1990). Examples of this include birds decreasing nest visitations in response to nest predators (Ghalambor & Martin, 2002), restricting movement in high risk areas (Rodriguez, Andren & Jansson, 2001), and changing nest-site locations to lower risk areas (Forstmeier & Weiss, 2004). Indeed, predation risk has been identified as an influential evolutionary mechanism in passerine life-history characteristics (Martin, 1995). Nest predation risk can also interact with other selection pressures, such as brood parasitism (Krüger, 2004) or microclimate (Tieleman, Van Noordwijk & Williams, 2008), through directional or stabilizing selection (Krüger, 2007), which can ultimately affect species’ nest-site selection behaviors. Exploring variation in nest survival probabilities across environmental gradients, and how behavioral plasticity affects those relationships, adds to our understanding of the evolutionary ecology of nesting and provides potential insight into habitat management considerations for species of conservation concern.

Gray vireos (*Vireo vicinior*) are a species of conservation concern at state and federal levels (U.S. Fish and Wildlife Service [USFWS], 2008; New Mexico Department of Game & Fish, 2018) that is most commonly found in pinyon (*Pinus* spp.)-juniper (*Juniperus* spp.) habitats, such as juniper savannas (Harris, Smith & McMurry, 2020) and pinyon-juniper shrublands (Schlossberg, 2006) throughout the species’ breeding range (Barlow, Leckie & Baril, 2020). However, little is known about the species’ habitat requirements in pinyon-juniper habitats. In the southwestern United States, gray vireos often rely on junipers as a nesting substrate (Harris, Smith & McMurry, 2020) and are more frequently found in areas with high proportions of juniper, lower proportions of pinyon pines, and greater shrub density (Schlossberg, 2006). Consequently, gray vireo demography is likely dependent on the availability and quality of pinyon-juniper habitats. Although data from the Breeding Bird Survey suggests that overall population size has remained stable (Sauer et al., 2017; Pardieck et al., 2018), the species may be susceptible to future declines due to habitat loss (Pierce, 2007), particularly under future climate change scenarios (Gardali et al., 2012). Additionally, population stability varies considerably by state. For example, populations in California have declined by 75–95% (Hargrove & Unitt, 2014), while populations in New Mexico have increased (Sauer et al., 2017; Pardieck et al., 2018).

Nest survival for species in the Vireonidae family is highly dependent on brown-headed cowbird (*Molothrus ater*) parasitism rates, typically resulting in relatively low reproductive output compared to other passerines (Barber & Martin, 1997; Woodworth, 1997; Smith, Reynolds & LeBuhn, 2005; Hargrove & Unitt, 2017). Brown-headed cowbird parasitism has been shown to occur at 43–93% of black-capped vireo (*Vireo atricapilla*) nests (Graber, 1961; Grzybowski, 1991), 43–75% of warbling vireo (*Vireo gilvus*) nests (Gardali & Ballard, 2000), and 49% of white-eyed vireo (*Vireo griseus*) nests (Hopp, Kirby & Boone, 1995). One possible explanation for high parasitism rates across the Vireonidae family is that most species of vireos nest on the periphery of the nest substrate, often on a terminal fork (Bent, 1965). This may increase their visibility and consequently their susceptibility to brood parasitism and predation from avian predators (Liebezeit & George, 2002).

Gray vireo nest survival likely varies by region and habitat. In central New Mexico, apparent nest survival ranged between 37–60%, although these estimates did not account for nest exposure time (DeLong & Williams, 2006; Frei & Finley, 2008; Walker & Doster, 2009; McMurry, Smith & Harris, 2019). One report from central New Mexico estimated daily nest survival probabilities of 59 gray vireo nests in pinyon-juniper woodlands, and found that mean nest survival was 0.269, with predation as the primary cause of nest failure (approximately 58% of nest failures) (Wickersham & Wickersham, 2015). In contrast, populations in southern California nesting in arid shrub-dominated chapparal have relatively poor nest survival (0.08), with high levels of nest predation (83% of failures) and relatively low levels of parasitism by brown-headed cowbirds (13% of failures) (Hargrove & Unitt, 2017). The most common nest predator in this region was the California scrub jay, which accounted for 67% of predation events. Nest survival probabilities were best explained by a negative effect of surrounding shrub height and a positive effect of nest height (Hargrove & Unitt, 2017). This variation in nest survival probabilities by region and habitat may be the result of differing predator communities or nest-site characteristics associated with vegetation communities (DeGregorio et al., 2016). For example, nests located in junipers found in New Mexico may provide thicker cover and greater concealment than the shrubs of southern California, resulting in less predation from avian predators, which tend to rely on visual cues (Colombelli-Negrel & Kleindorfer, 2009; Latif, Heath & Rotenberry, 2011). Additionally, such regional differences in predation risk, could interact with nest placement characteristics, such as nest height. For example, DeGregorio et al. (2016) showed that taller nests are generally more likely to be depredated by corvids and rodents, and that predation by corvids is more likely in the Southwest.

We estimated gray vireo daily nest survival rates to determine how characteristics of junipers and nest placement strategies may influence daily nest survival probabilities. We also sought to identify gray vireo habitat management strategies for pinyon-juniper habitats. Based on results from Hargrove & Unitt (2017), we hypothesized that daily nest survival would be driven by nest height and the height of the surrounding vegetation. Specifically, we were interested in advancing this finding to determine if the survival probabilities across various nest heights was dependent on the height of surrounding vegetation. We also expected nest survival to be negatively related to tree density, as denser woodland cover may be more suitable for nest predators, such as Woodhouse’s scrub jay (*Aphelocoma woodhouseii*) (Curry et al., 2017).

## Materials & methods

### Study site

**Figure 1:**
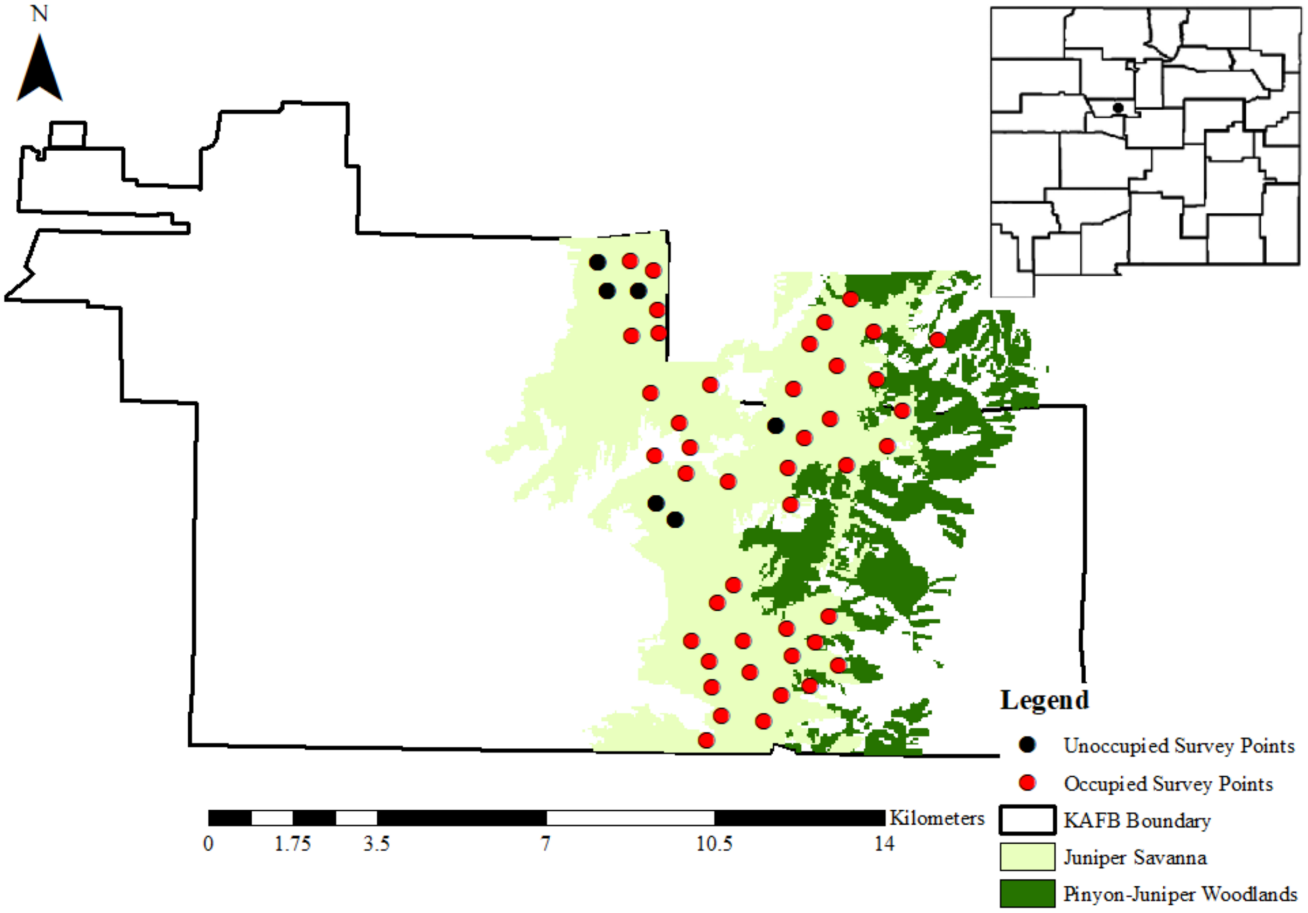
The location of Kirtland Air Force Base in Albuquerque, New Mexico. Kirtland Air Force Base is represented as a black dot on the map of New Mexico (USA) in the top-right corner. Pinyon-juniper woodlands and juniper savannahs are shown in dark green and light green, respectively. Black and red dots represent the 50 random points that were surveyed for gray vireos in 2016–2018, where black dots are points where no gray vireos were detected and red dots are points where at least 1 gray vireo was detected between 2016–2018. Data for cover type distributions were provided by Kirtland Air Force Base personnel.

We located gray vireo nests on Kirtland Air Force Base (KAFB), which occupies approximately 21,000 ha south of Albuquerque, NM, USA (Fig. 1), from May–August in 2016–2018. Weather patterns during this time period were consistent with the general climate of the region. Temperatures from May–August are generally 18.3–25.7 °C, with a mean monthly precipitation total of 25.9 mm (Western Regional Climate Center 1914–2012). Albuquerque’s wet season is primarily July–September, when approximately 40% of the annual precipitation occurs (Gutzler and Nims, 2005). Elevation on KAFB ranges from 1,600 to almost 2,400 m and encompasses four primary landcover types following an elevation gradient: grasslands, persistent pinyon-juniper woodlands, ponderosa pine (*Pinus ponderosa*) woodlands, and wetlands/arroyos (U.S. Department of Defense, 2012). We focused our surveys in areas where junipers predominated over pinyon pines, such as transition zones between grasslands and persistent pinyon-juniper woodlands. These areas were categorized as juniper savannas by Kirtland Air Force Base contractors (Johnson, Smith & Petersen, 2013) (Fig. 1) and were primarily composed of one-seed juniper (*Juniperus monosperma*), four-winged saltbush (*Atriplex canescens*), sand sagebrush (*Artemisia filifolia*), rubber rabbitbrush (*Ericameria nauseosa*), and grama grasses (*Bouteloua* spp.) (U.S. Department of Defense, 2012). Access to Kirtland Air Force Base was provided by the U.S. Air Force (Kirtland Air Force Base Pass ID number: 9748-46667474).

### Nest searching

To identify breeding territory locations, we conducted two rounds of call-back surveys for gray vireos at 50 random locations from 1 May–15 June in 2016–2018. We used ArcGIS (v. 10.2.2) to create 50 random locations ≥ 500 m apart in areas designated as juniper savannas by KAFB contractors (Johnson, Smith & Petersen, 2013). Elevations at random locations ranged from 1823–2148 m. Surveys were conducted at the same random points each year. At each point, we conducted a 10-min survey consisting of 4 min of passive surveying, followed by 1 min of broadcast of conspecific vocalizations from a portable speaker (Cass Creek Big Horn), followed by 5 min of passive surveying (Harris, Smith & McMurry, 2020). Broadcasts were of an adult male Gray Vireo song from the Arizona Field Ornithologist Sound Library and were played at maximum volume. There was no maximum distance for recording gray vireo observations since the objective of surveys was just to locate breeding territories for nest searching. We also located additional territories opportunistically when traveling between points.

Once a breeding territory had been located, we searched for nests by following individuals exhibiting nesting behaviors (*e.g*., carrying nesting material, males singing from nests, etc.). Territories were visited for nest searching approximately 3–5 times per week until a nest was found. There was no standardized amount of time spent nest searching at each territory, as this was dependent on gray vireo behavior and weather conditions. During a visit, we stopped nest searching if gray vireos were not exhibiting behavior indicative of nest building or incubation (*e.g*., males and females foraging together) or if there were adverse weather conditions (*e.g*., high winds, rain) then we stopped nest searching; however, neither behavioral data nor weather data were recorded during nest searching. When a nest was found, we estimated the date that the first egg was laid using the incubation period (12–14 days, Barlow, Leckie & Baril, 2020) and the nestling duration (13–14 days, Barlow, Leckie & Baril, 2020). Nests were monitored an average of once every four days (range: 1–20 days) to determine survival. When possible, we determined the status of nests using binoculars from 5–10 m away to minimize the impact of monitoring on nest outcome (Martin & Geupel, 1993). We categorized the status of the nest at each visit as active, successful, depredated, parasitized, or abandoned. Depredated nests were identified by broken or missing eggs or nestlings, and nests were considered “successful” if ≥1 nestling fledged. Successful nests were confirmed by finding fledglings, which frequently vocalized near the nest location shortly after fledging. Nests that we were unable to determine the fate of were excluded from analysis. All methods were approved by the United States Geological Survey (permit number 21774), New Mexico Department of Game and Fish (Permit number 3623), and Oklahoma State University Institutional Animal Care and Use Committee (IACUC, AS-15-16).

### Nest placement and vegetation surveys

We were interested in how daily nest survival probability was affected by nest placement, characteristics of the nesting tree, and characteristics of the surrounding habitat. Thus, we collected data at three scales: the nest, the nesting tree, and a 25-m radius around the nesting tree. A 25-m radius scale was chosen because it is relevant for nest defense behavior (*e.g*., alarm calling) (Bates, 1992). For nest placement, we measured height from the ground, distance from the edge of the nest tree (hereafter: distance from edge), and orientation of the nest from the central trunk (Smith, Reynolds & LeBuhn, 2005). Distance from edge was measured as the distance from the outward-facing side of a nest to the farthest foliated edge on the same side of the nesting tree. Nest orientation was defined as the compass azimuth of the nest relative to the center of the tree. For example, a nest that was located on the south side of a nesting tree would have an orientation of 180°. For the nesting tree and all trees within a 25-m radius around the nest, we measured height (m ± 1 cm), width of foliage (m ± 1 cm) measured at its widest point, and an estimation of foliage density for junipers. Foliage density was only considered for junipers because gray vireos only nested in junipers at our study site. Foliage density has been used as a proxy for nest concealment in other studies (Banks & Martin, 2001; Borgmann & Conway, 2015). To estimate foliage density for many trees expediently, we used a modified Braun-Blanquet method (Wikum & Shanholtzer, 1978; Harris, Smith & McMurry, 2020), where we estimated the percentage of limbs and trunks that were obscured by foliage at the side of the tree that the nest was located on and assigned a categorical value: 1 (0–25%), 2 (26–50%), 3 (51–75%), or 4 (76–100%). For measurements at the 25-m radius scale, we determined the mean tree height and width for trees within the plot, and counted the total number of junipers within this scale.

## Statistical analyses

We estimated daily nest survival probabilities (*i.e*., the probability a nest survives 1 day, S_d_) using logistic exposure models (LEMs) (Shaffer, 2004). The dependent variable for all LEMs was a binary nest survival variable for each nest check, independent variables included characteristics related to nest placement and the surrounding vegetation, and all models included the year as a random effect. We did not account for territory ID within year as a source of lack-of-independence because not all territories were color-banded. Consequently, territories that renested after failure may be a source of lack-of-independence in our samples. We used the glmer function in the lme4 package to create our LEMs in Program R (v. 3.5.1) and evaluated models using Akaike Information Criterion corrected for small sample size (AICc) (Akaike, 1973, Burnham & Anderson, 2002). The glmer function in lme4 allows the model to incorporate a custom log-link function that contains an exponent of one over time, as needed in LEMs (Shaffer, 2004).

We developed a global model with all additive combinations of variables. Interactions were also included to determine if parameters related to nest placement (*e.g*., nest height, distance from the edge) could mitigate vegetation characteristics that may have lower nest survival probabilities (*e.g*., low foliage density) or to test the hypothesis that a negative interaction between nest height and the height of surrounding vegetation positively influences nest survival. Models with interacting parameters also included additive effects of those parameters. Independent variables with pairwise correlation coefficients (|r|) > 0.7 were not included within the global model (Dormann et al., 2013) to prevent inflated variances (and consequently standard errors) of parameters, which decreases the reliability of identifying relevant predictors. Tree heights were highly correlated with tree widths so only tree heights were incorporated in the global model, for a total of seven independent variables. Preliminary plotting suggested that the relationship between nest survival and juniper count was unimodal, so we also included a quadratic (squared) term for juniper count in the global model. We then dredged the global model using the MuMIn package (v. 1.43.6), which produces an AICc value for every possible additive combination of variables and interaction terms (Doherty, White & Burnham, 2012). We selected models for the final model subset if they had a ΔAICc ≤ 2.0 (Burnham & Anderson, 2002), did not include uninformative parameters as described by Arnold (2010) and Leroux (2019), and if 95% confidence intervals of the parameter estimates did not overlap with zero. Coefficients from models included in the final model subset were averaged (Burnham & Anderson, 2002) to estimate the S_d_, where mean values of independent variables were multiplied by model-averaged coefficients (Shaffer, 2004). We estimated cumulative nest survival (*i.e*., the probability a nest fledges ≥ 1 young, S_c_) by raising S_d_ to 32.4, which was the mean cumulative number of days of egg laying, incubation, and nestling stages of Gray Vireos in our study. We estimated the sampling variance of S_c_ using the delta methods (Powell, 2007).

## Results

The earliest detection of gray vireos occurred on 19 April 2017. We detected gray vireos at 56% (*n* = 28) of random points in 2016, 64% (*n* = 32) of points in 2017, and 58% (*n* = 29) of points in 2018. The first nest each year was found on an average start date of 12 May. All nests were located in one-seed juniper. Mean nest height was 2.2 ± 0.7 m (SD) above the ground and mean distance from edge was 0.67 ± 0.53 m. Gray vireos nested on all sides of nesting trees (north: 33%, south: 29%, east: 20%, west: 18%). Mean nest-tree height was 3.5 ± 0.97 m and the mode foliage density around nests 50–75%.

We monitored 89 gray vireo nests from 2016–2018. Fifty-five nests fledged ≥ 1 young (Table 1). The most common cause of nest failure was predation, which accounted for 62% of nest failures (*n* = 21). Brood parasitism by brown-headed cowbirds accounted for 24% of failures (*n* = 8) and the remainder of nests were abandoned (*n* = 5) (Table 1). All parasitized nests were subsequently abandoned.

**Table 1:** Summaries of gray vireo nest outcomes on Kirtland Air Force Base in Albuquerque, New Mexico, USA from 2016–2018.

	2016		2017		2018		Total	
Nest outcome	*N*	Ratio	*N*	Ratio	*N*	Ratio	*N*	Ratio
Fledged ≥ 1	12	0.48	30	0.73	13	0.57	55	0.62
Depredated	5	0.20	8	0.20	8	0.35	21	0.24
Parasitized	4	0.16	3	0.07	1	0.04	8	0.9
Abandoned*	4	0.16	0	0	1	0.04	5	0.06
Total	25		41		23		89	

**Notes:**

* Abandoned nests are not including parasitized nests that were subsequently abandoned.

Nests were monitored on average once every four days to determine nest fate.

**Figure 2:**
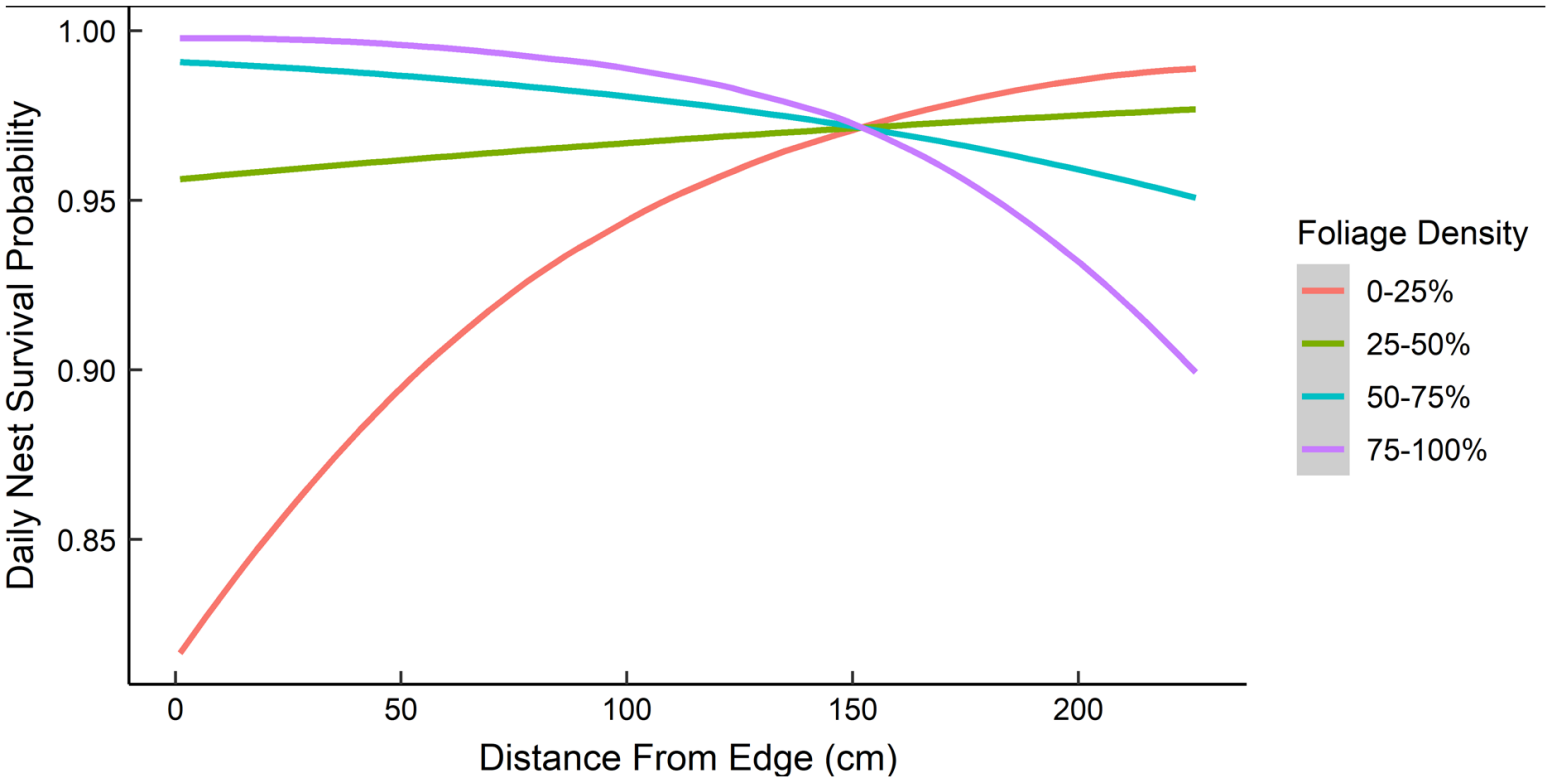
The predicted daily nest survival probabilities for gray vireo nests as a function of juniper foliage density and distance to edge. Daily nest survival probabilities were estimated as the probability that at least one nestling survived one day. Foliage density was measured as the percentage of woody stems obscured by foliage around the nest. Distance from edge was measured as the distance (cm) from the outward-facing side of a nest to the farthest foliated edge on the same side of the nesting tree. Data are from 89 gray vireo nests found on Kirtland Air Force Base in Albuquerque, New Mexico, USA from 2016–2018.

**Figure 3:**
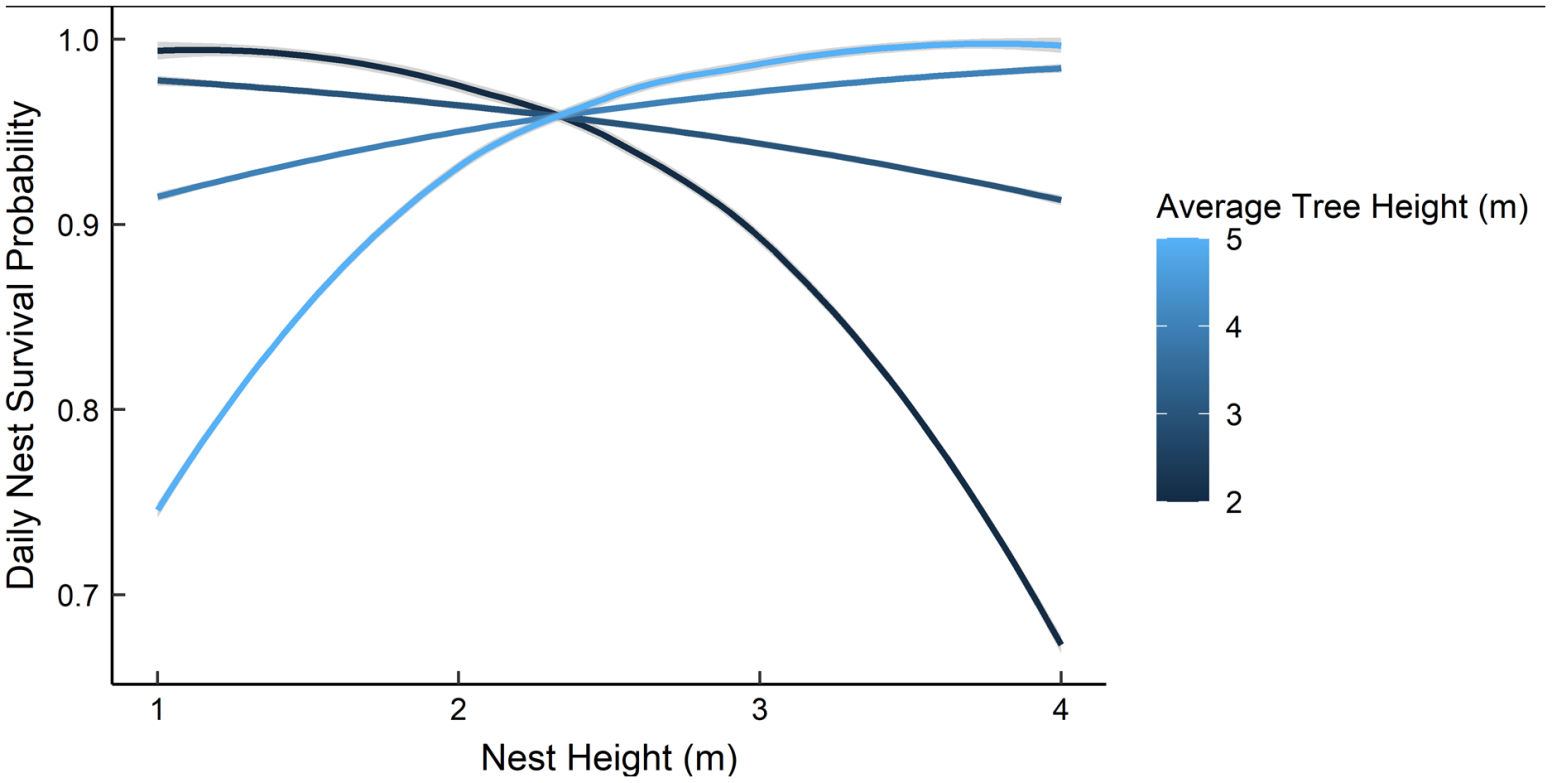
The predicted daily nest survival probabilities for gray vireo nests as a function of nest height and the height of the surrounding trees. Daily nest survival probabilities were estimated as the probability that at least one nestling survived one day. We predicted a negative interaction between nest height and the height of surrounding vegetation. However, we found evidence for a positive interaction between these variables. Data are from 89 gray vireo nests found on Kirtland Air Force Base in Albuquerque, New Mexico, USA from 2016–2018.

Dredging a global model produced seven models with a ΔAICc ≤ 2.0 (Table 2). All models included an interaction of foliage density and distance to edge. The two models representing our two hypotheses (*i.e*., an interaction between nest height and the height of surrounding vegetation, and juniper count), both had a ΔAICc ≤ 2.0. However, the 95% confidence intervals for juniper count overlapped with zero. After removing models with uninformative parameters and parameters with 95% confidence intervals that overlapped with zero, there were two models that we used for statistical inference and model averaging (Arnold, 2010, Leroux, 2019) (Table 3). The top model was an interaction of foliage density and distance from edge (Table 3). Nest survival probabilities increased with foliage density when nests were close to the edge of nesting tree, and increased with distance from the edge of the nesting tree when foliage density was low (Fig. 2). Although the second best model was a more complex version of our top model, the parameters included were considered informative because it fell within two AICc units of the top model, included three extra parameters as opposed to one (Table 3), and the interaction included confidence intervals that did not overlap with zero (Leroux, 2019). The second model included an interaction between nest height and the average height of surrounding trees, which represented our first hypothesis. However, we found a positive interaction where taller nests were more likely to survive if surrounded by taller trees and shorter nests were more likely to survive when surrounded by shorter trees (Fig. 3). We used model averaged coefficients from these models to estimate S_d_ and S_c_ as 0.983 (95% CL [0.973–0.989]) and 0.573 (95% CL [0.444–0.702]), respectively (Table 2).

**Table 2:** Logistic exposure models of gray vireo daily nest survival probabilities.

Model	*K*	ΔAICc	ω_i_	Deviance
^a^FolDen * DistEdg	5	0	0.08	197.2
FolDen * DistEdg + NestHt * TrHt_avg_	8	0.6	0.06	191.5
FolDen * DistEdg + NestHt	6	0.7	0.06	195.8
FolDen * DistEdg + JunCt + JunCt^2^	7	1.2	0.05	194.2
FolDen * DistEdg + NeTrHt	5	1.3	0.05	196.4
FolDen * DistEdg + JunCt + JunCt^2^ + NeTrHt	8	1.7	0.04	192.6
FolDen * DistEdg + TrHt_avg_	6	1.9	0.03	197.0
Null model	2	13.0	0.00	216.3

**Notes:**

a AICc value of 207.2.

Covariate descriptions: FolDen, foliage density of the nesting tree; DistEdg, distance of the nest from the edge of the nesting tree; NestHt, nest height; FolDen_avg_, the average foliage density of junipers in the nesting plot; TrHt_avg_, the average height of trees in the nesting plot; JunCt, juniper count in the nesting plot; NeTrHt, nest tree height. Relative model performance was evaluated using Akaike Information Criterion corrected for small sample sizes (AICc), AICc weight (ω_i_), and Deviance as a goodness-of-fit metric. *K* is the number of parameters in the model, including an error term and a random intercept. Models were formed from 89 nests found from 2016–2018 on Kirtland Air Force Base in Albuquerque, New Mexico, USA. Only models with ΔAICc ≤ 2.0 and a null model are included.

**Table 3:** The parameters included in our two competitive models of gray vireo daily nest survival probabilities.

Model parameters	β	Odds ratio	Standard error	95% CI
FolDen * DistEdg				
Distance from edge	−0.27	0.76	0.16	[−0.58, 0.06]
Foliage density	0.74	2.1	0.16	[0.35, 1.16]
Foliage density * Distance to edge	−0.45	0.64	0.17	[−0.79, −0.13]
FolDen * DistEdg + NestHt * TrHt_avg_				
Distance from edge	−0.25	0.78	0.17	[−0.59, 0.10]
Foliage density	0.85	2.35	0.22	[0.42, 1.32]
Foliage density * Distance to edge	−0.50	0.60	0.17	[−0.85, −0.18]
Nest height	−0.34	0.71	0.20	[−0.74, 0.04]
Average tree height	−0.11	0.90	0.18	[−0.45, 0.26]
Nest height * Average tree height	0.37	1.45	0.18	[0.02, 0.73]

**Note:**

Each parameters includes beta coefficients (β), odds ratios, standard errors, and 95% confidence intervals. The parameters of each of the top models are indented under the model name. Models were formed using data from 89 gray vireo nests found in 2016–2018 on Kirtland Air Force Base in Albuquerque, New Mexico, USA.

## Discussion

The best predictor of gray vireo nest survival was a negative interaction of foliage density of junipers with distance from edge, suggesting that gray vireos could mitigate predation and parasitism risk from low visual concealment through variation in fine-scale nest placement. Nests were more likely to survive if they were located in trees with high foliage density and placed close to the edge of the nesting tree, or if they were placed closer to the interior of nesting trees when located in trees with poor foliage density. This result is in consonance with previous studies on behavioral plasticity of birds as a mechanism to increase nest survival probabilities (Sockman, 1997; Forstmeier & Weiss, 2004). The variation in distance to edge at our study site (σ = 0.53 m, range = 0.0–2.5 m) was greater than what was observed in the Sacramento Mountains of New Mexico (σ = 0.09 m, *n* = 13) (Britt & Lundblad, 2008) but was similar to what was observed by Wickersham & Wickersham (2015) at our study site (range = 0.0–2.2 m, *n* = 63). However, we did not find evidence that gray vireos preferentially placed nests closer to the interior of trees when foliage density was low, as we did not find a negative correlation between distance to edge and foliage density (*r* = 0.02). Consequently, it is unclear if this pattern is simply random variation resulting in a positive effect on survival or if it represents a meaningful response of evolutionary selection pressures favoring behavioral plasticity.

Nesting closer to the interior of the nesting tree when foliage density is low would likely increase concealment from avian predators and brood parasites, but may increase risk from ground-dwelling predators (Colombelli-Negrel & Kleindorfer, 2009). Indeed, the singular effect of distance to edge (without considering foliage density) had a negative effect on daily nest survival, possibly due to increased predation from ground-dwelling predators. Visual concealment of nests has been shown to be particularly correlated with predation by other avian species (Santisteban, Sieving & Avery, 2002; Colombelli-Negrel & Kleindorfer, 2009, Latif, Heath & Rotenberry, 2011) and brood parasitism by brown-headed cowbirds (Larison et al. 1998). It is unclear what the most common nest predator was at our study site. However, California scrub jays were the most common nest predator of gray vireos in California (Hargrove & Unitt, 2017), and Woodhouse’s scrub jays were abundant and frequently observed in gray vireo territories at our study site. Although nest concealment has been linked to nest survival and predation risk for a variety of other passerine species (Martin & Geupel, 1993; Davis, 2005; Latif, Heath & Rotenberry, 2011), concealment may be more critical for species in the Vireonidae family because most species nest on the periphery of their nesting substrate (Bent, 1965).

Based on Hargrove & Unitt (2017) results, we hypothesized that daily nest survival would be influenced by the interaction of nest height and the height of surrounding vegetation; specifically, we expected that tall nests would have higher survival if they were surrounded by shorter vegetation due to decreased scrub jay use in areas where vegetation was shorter and sparser. We tested this by incorporating a model with an interaction between nest height and mean tree height and a model with juniper count; we found a positive interaction between nest height and mean tree height, and a unimodal response to juniper count. Hargrove & Unitt (2017) hypothesized that taller surrounding vegetation may provide better perches for predators and brown-headed cowbirds, which may decrease nest survival. At our study site, gray vireos predominately nested in juniper savannas, where Woodhouse’s scrub jays and brown-headed cowbirds were commonly observed and occurrence was anecdotally uniform. Several studies have shown that taller nests are more likely to be depredated by avian predators and squirrels (Colombelli-Negrel & Kleindorfer, 2009, DeGregorio et al., 2016), which are presumed to be more prevalent in taller vegetation. Consequently, it is unclear why taller nests would be more likely to survive when surrounded by taller vegetation. Wickersham & Wickersham (2015) suggest that taller nests may provide better vantage points when surrounded by taller vegetation; however, the relationship between nest height and the height of surrounding vegetation warrants further exploration. Additionally, our data suggested that optimum juniper density for nest survival was 15–20% of the available cover; however, the confidence intervals for this relationship overlapped with zero. It is interesting to note, however, that this percentage of juniper cover is similar to optimum juniper density for gray vireo nest-site selection (Harris, Smith & McMurry, 2020).

Brown-headed cowbird parasitism accounted for 26% of nest failures, which is more than what was observed in California (13%, Hargrove & Unitt, 2017). In New Mexico, brown-headed cowbirds have been shown to utilize pinyon-juniper woodlands in the morning hours and grassland habitats in the afternoons, particularly when breeding (Goguen & Matthews, 2001). In our study, gray vireos more commonly nested in juniper savannas, where juniper density was 15–30% at a 25-m radius scale (Harris, Smith & McMurry, 2020). This transition zone between persistent pinyon-juniper woodland and desert grassland may have greater parasitism risk, as sparse stands of junipers provide necessary perches for females to locate nests (Lowther, 1993) and grasslands provide foraging habitat (Goguen & Matthews, 2001). However, throughout their range gray vireos seem less susceptible to brood parasitism than other species in Vireonidae (Hargrove & Unitt, 2017; Barlow, Leckie & Baril, 2020), such as black-capped vireos (Campomizzi et al., 2013) and least Bell’s vireo (Kus, 1999). This may be because gray vireos seem to be capable of identifying brown-headed cowbird eggs and abandoning parasitized nests (Barlow, Leckie & Baril, 2020). All of the parasitized gray vireo nests observed in our study were subsequently abandoned, which is consistent with other observations (Barlow, Leckie & Baril, 2020). Although this ability to reject brown-headed cowbird eggs may make gray vireos a less desirable host species, more data are needed on the effect of brood parasitism on gray vireo productivity throughout the species’ range.

Cumulative nest survival probabilities of gray vireos at our study site were higher than what has previously been observed (Hargrove & Unitt, 2017). Hargrove & Unitt (2017) found that daily nest survival and cumulative nest survival in chaparral habitat of California was 0.91 and 0.08, respectively. We found daily and cumulative nest survival was 0.983 (95% CI [0.973–0.989]) and 0.573 (95% CI [0.444–0.702]), respectively. Gray vireo populations throughout California occur in small, isolated populations (Hargrove & Unitt, 2014). Low nest survival in these regions is likely the primary factor causing low densities (Hargrove & Unitt, 2017). Populations at our study site occurred in large patches of juniper savannas. Unpublished reports suggest that gray vireos in pinyon-juniper habitat tend to have higher nest survival (Barlow, Leckie & Baril, 2020; Wickersham & Wickersham, 2015; McMurry, Smith & Harris, 2019) than what was shown in chaparral (Hargrove & Unitt, 2017). These higher survival probabilities were mostly the result of lower predation rates at our study site (24%) than populations in California (68%) (Hargrove & Unitt, 2017). Previous work on gray vireo nest-site selection at our study site has shown that gray vireos select junipers with higher foliage density than random locations (Harris, Smith & McMurry, 2020), suggesting that nest sites are in locations with the greatest amount of concealment. The differences in predation risk between the two locations may also be a function of differing predator communities between the two habitat types. However, more data are needed on common nest predators for gray vireo nests in pinyon-juniper habitats.

## Conclusions

We found that daily nest survival for gray vireos was related to nest placement and the foliage density of nesting trees. Nests were more likely to survive when in trees with high foliage density or when they were placed closer to the interior of trees when foliage density was low. These results add to our understanding of the evolutionary ecology of an understudied species, while providing usable results on nest survival probabilities that could aid in the conservation of gray vireos.

Future habitat management strategies may seek to remove poorly foliated junipers in areas where juniper densities are high. Gray vireos have been shown to select nest sites where juniper density represents 15–30% of the available cover, and rarely select areas with densities greater than 50% (Harris, Smith & McMurry, 2020). In areas where juniper density is greater than 50% of the available cover, thinning of poorly foliated junipers may increase the amount of available nesting habitat while also increasing habitat quality.

## Supplemental Information

10.7717/peerj.12477/supp-1 Supplemental Information 1 Code for logistic exposure models and creating figures 1 and 2. Code was constructed in RStudio (v. 1.0.153) and R (v. 3.5.1) to construct logistic exposure models, evaluate model performance, and create figures.

10.7717/peerj.12477/supp-2 Supplemental Information 2 Daily Nest Survival Data. Each data point represents a nest-checking event at a Gray Vireo nest. All nests were found on Kirtland Air Force Base in Albuquerque, New Mexico in 2016–2018.

## Additional Information and Declarations

### Field Study Permissions

The following information was supplied relating to field study approvals (*i.e*., approving body and any reference numbers): The United States Air Force approved this research (Kirtland Air Force Base Pass ID number: 9748-46667474).

### Data Availability

The following information was supplied regarding data availability: The raw data on Gray Vireo nest checks with associated independent variables and an R file of the code for running logistic exposure models are available in the Supplemental Files.
